# Health related quality of life among the below poverty line population in Bangladesh: A cross-sectional study

**DOI:** 10.1371/journal.pgph.0005309

**Published:** 2025-10-13

**Authors:** Md. Zahid Hasan, Khadija Islam Tisha, Kamrun Nahar, Mohammad Wahid Ahmed, Sayem Ahmed

**Affiliations:** 1 Health Economics and Financing, Health Systems and Population Studies Division, icddr, b, Dhaka, Bangladesh; 2 Nuffield Centre for International Health and Development, University of Leeds, Leeds, United Kingdom; 3 Department of Health Sciences, Brunel University London, Uxbridge, Middlesex, United Kingdom; PLOS: Public Library of Science, UNITED STATES OF AMERICA

## Abstract

The below-poverty-line (BPL) population experiences challenges in accessing quality healthcare services due to their limited affordability and vulnerability to illness. This paper assessed their health-related quality of life (HRQoL) and its associated determinants. A cross-sectional survey was conducted among 803 BPL individuals aged 18 and above in Tangail district from July to September 2018. To measure HRQoL, we employed a Bengali version of the EuroQol-5 dimensions (EQ-5D) questionnaire and the Visual Analogue Scale (VAS). Tobit regression models were applied to determine the association between HRQoL scores and sociodemographic characteristics, multimorbidity, and healthcare utilization in the last three months. The BPL population had an average EQ-5D score of 0.738 (SD = 0.192) and an EQ-VAS score of 0.750 (SD = 0.149). Among the five EQ-5D domains, 68% of the respondents reported experiencing some or extreme problems in the anxiety/depression dimension, and 55% reported pain/discomfort. The EQ-5D and EQ-VAS scores were significantly lower among individuals aged 35 years and above compared to those aged 18–34 years. Individuals with multimorbidity had significantly lower EQ-5D and EQ-VAS scores, by 0.250 and 0.158 (p < 0.001), respectively, compared to those without any health problems. Respondents in the richest asset quintile had significantly higher EQ-5D scores than those in the poorest quintile. The findings shed light on the keydeterminants of HRQoL among the BPL population in Bangladesh, such as age, multimorbidity, marital status, and wealth status. These determinants may help policymakers in developing interventions for improving the health and well-being of the BPL population. Tailored interventions are needed to address their specific needs for improving HRQoL, such as community-based programs for the older adults, provision of integrated healthcare for addressing multimorbidity, effective implementation of the current health protection scheme for the poor and other vulnerable groups, including widowed/separated individuals.

## Background

Over the last two decades, Bangladesh has made significant progress in poverty reduction from 48.9% in 2000 to 18.7% in 2022 [1]. Despite such progress, the country still faces substantial challenges, as approximately one-fifth of its population lives below the poverty line (BPL) [2]. This underprivileged community often struggles to meet their basic needs of life, such as access to quality healthcare, decent housing, clean water, and nutritious food [3]. Limited access to basic needs of life often makes people vulnerable to diseases, including infections, malnutrition, and chronic illnesses [4]. Moreover, despite being a fundamental human right, healthcare remains inaccessible to many individuals in low- and middle-income countries (LMICs), including Bangladesh [5]. As a result, many people are left without the necessary healthcare services, which potentially exacerbates their health problems and reduces their health-related quality of life (HRQoL). In LMICs, out-of-pocket expenditure (OOPE) is approximately 35.25% of the total healthcare expenditure, which is a significant barrier to accessing and utilizing healthcare services [6]. According to Bangladesh National Health Accounts, the OOPE for healthcare is approximately 68.5%, which is the highest among neighbouring countries [7]. The high OOPE amplifies the existing inequity in accessing and utilizing healthcare services, especially among poor people [5].

In recent years, HRQoL has emerged as an integral part of public health surveillance. This is because self-assessed health status has proven to be a more accurate predictor of future mortality and morbidity compared to various objective measures of health [8,9]. HRQoL reflects one’s subjective state of health and well-being and includes mental, physical, and social functioning capacity [10]. Measuring HRQoL may help to identify vulnerable populations for targeted interventions to improve their HRQoL and avert deterioration in health-related outcomes. Such estimates may be used for a wide variety of purposes, including but not limited to designing health-related interventions, allocating funds to address gaps, formulating successful strategies, and tracking the effectiveness of healthcare programs [11,12].

To date, several studies in LMICs have assessed HRQoL among different subgroups of populations [13,14] and for specific disease conditions [15,16]. Studies conducted in China, Singapore, Korea, and Greece found that demographic and socioeconomic factors, such as low socioeconomic status, were linked with lower HRQoL [17–20]. Another study conducted in China revealed that individuals from low-income families had poorer HRQoL than the general population [21]. However, to the best of our knowledge, little is known about HRQoL among the BPL population in Bangladesh. Information on baseline HRQoL is essential for designing, implementing and tracking outcomes of public health interventions aiming to improve the health and wellbeing of the community. Therefore, this study aimed to evaluate the HRQoL of the BPL population in a subdistrict of Tangail district of Bangladesh.

## Methods

### Ethics statement

This study was approved by the Research Review Committee and the Ethical Review Committee of the International Centre for Diarrhoeal Disease Research, Bangladesh (icddr,b). Participants in the study provided written informed consent, and their participation was voluntary.

### Inclusivity in global research

Additional information regarding the ethical, cultural, and scientific considerations specific to inclusivity in global research is provided in the Supporting Information (S1 Checklist).

### Study population and sample

A cross-sectional study was conducted among the BPL population in Kalihati Upazila (subdistrict) of Tangail district. This site was purposively selected because it was the implementation site of the health protection scheme of the government of Bangladesh called Shyastho Suroksha Karmasuchi (SSK), which specifically identifies the BPL population. In Kalihati Upazila, the total number of households is 89,351, of which 35,740 (40%) households were identified as BPL under the scheme [22]. If a household meets at least two of the following three criteria, the household was listed as a BPL household; a) the head of the family was a regular day laborer; b) the household had no land other than their homestead; and c) the household had no permanent or stable income sources [22]. A total of 828 households were selected for the survey from Kalihati Upazila (details of sample size estimation have been described elsewhere) [23]. In this Upazila, there are 15 Unions (each representing a group of villages) and the enrolment of BPL households varied across the 15 Unions. Before conducting the survey, a list of BPL households was collected from the scheme authority. Out of 828 households, the number to be surveyed from each union was determined and selected in two phases. In the first phase, the number of BPL households to be surveyed from a union was determined based on the proportion of total BPL households in that specific Union. In the second phase, a simple random sampling technique was used to select the required number of BPL households. From each household, the household head or an adult member, in the absence of the household head, was selected for the interview. Among the 828 selected respondents, 806 responded to the survey (response rate: 97%). However, three participants provided incomplete responses and were excluded from the final analysis.

### Measurement of HRQoL

We assessed HRQoL using an adapted Bangali version of the EQ-5D-3L tool. The EQ-5D-3L questionnaire was originally developed by European investigators in 1990 [24]. HRQoL data are self-reported, and the tool has been widely applied to assess health outcomes across diverse diseases and conditions [25,26]. This instrument evaluates five dimensions of health: mobility, self-care, usual activities, pain/discomfort, and anxiety/depression. Each dimension is measured on a three-level scale: no problems, some problems, and extreme problems [24]. The second part contains a self-reported visual analogue scale (VAS), which is 20 cm in length. To obtain health state scores, the scale has endpoints of 100 and 0, where 100 indicates the “best imaginable health state” and 0 indicates the “worst imaginable health state” [27,28]. The participants were asked to mark anywhere on the scale to indicate how they felt about their health state for the day. HRQoL was assessed using regional tariffs (Thailand) available for EQ-5D in the absence of such population norms for Bangladesh. We also assessed the internal consistency using Cronbach’s alpha and found an alpha score of 0.701, indicating an acceptable level of internal consistency [29].

### Data collection

Trained interviewers collected data through face-to-face interviews with the selected respondents between July and September 2018. The interviewers explained the study objectives to the respondents before starting the interview. Informed written consent was obtained from each participant. The Bengali version of the EQ-5D, along with the VAS instrument, was used to assess the HRQoL of the respondents [30]. We used a pretested, standardized questionnaire to collect information from respondents. The pretesting process ensured that the Bengali translation was understandable and appropriate for BPL participants, including those with low literacy levels. Feedback from pretesting was incorporated to refine the wording and improve clarity. In addition, the demographic and socioeconomic characteristics of the respondents were also collected (S1 Data).

### Data analysis

We conducted both descriptive and multiple regression analyses. We have presented the percentage of respondents reporting problems in each EQ-5D dimension by demographic and socioeconomic characteristics. Pearson’s chi-square test and two-sample tests on equality of proportions were conducted to assess the association between problems reported by the respondents and their demographic and socioeconomic characteristics. We conducted the Shapiro-Wilk test to assess the normality of the data distribution and found that the scores did not follow a normal distribution. Consequently, we used non-parametric tests, the rank-sum test for variables with two categories and the Kruskal-Wallis test for variables with more than two categories to measure the association between EQ-5D utility score, VAS scores, and characteristics of respondents. We classified the socioeconomic status of the respondents based on their reported possession of durable assets and applied principal component analysis (PCA). The PCA scores were generated using the possession of durable goods (e.g., mobile phones and televisions) [31]. Based on these scores, the respondents were divided into five equal groups (quintile), each representing 20% of the population.

We employed five separate multiple logistic regression models, one for each dimension of the EQ-5D, where experiencing a problem (some or extreme) was used as the dependent variable in dichotomous form (yes or no). In all the models, the respondent’s characteristics, such as sex, age, education, occupation, and income; illness, such as chronic illness and multimorbidity (having more than one illness); and healthcare utilization history, were included as independent variables. These models provided odds ratios as a measure of the association of the problem reported in an EQ-5D with the explanatory variables. Since the EQ-5D score and VAS score are censored variables with a maximum value of 1. We used two Tobit regression models separately to measure the association with respondents’ sociodemographic characteristics (e.g., age, sex, education, income) and illness and healthcare utilization related variables (e.g., health problems, healthcare seeking).

## Results

### Characteristics of the respondents

Table 1 presents the background characteristics of the respondents. The highest proportion of the respondents were aged between 18 and 34 years (34.6%), followed by 35–44 years (30.3%), 45–60 years (27.6%), and more than 60 years (7.5%). Most respondents were female (76.3%), and approximately 92.9% were married. Approximately 55.3% of the respondents reported having no formal education, 24.5% had received primary-level education, and 20.2% had secondary or higher level of education. The majority of respondents were housewives (64.6%), followed by agricultural workers (17.8%), small business owners (6.4%), and service workers (4.7%). Approximately 9% of the respondents reported suffering from chronic diseases in the last three months. More than 33% of the respondents reported having at least one health problem, and approximately 2% had multimorbidity (i.e., more than one health problem) in the three months prior to the survey. Approximately 27.4% of respondents sought healthcare for their illness in the last three months before the survey. Most of the surveyed respondents (53.1%) lived in households consisting of 4–5 persons, followed by households with fewer than four persons (37.1%), while a smaller percentage (9.8%) llived in households with more than six persons.

**Table 1:** Background characteristics of the respondents (n = 803).

Characteristics	n	Percentage (95% CI)
**Age group**		
18-34	278	34.6 (31.4-38.0)
35-44	243	30.3 (27.2-33.5)
45-60	222	27.6 (24.7-30.8)
60+	60	7.5 (5.8-9.5)
**Sex**		
Male	190	23.7 (20.8-26.7)
Female	613	76.3 (73.3-79.2)
**Marital status**		
Married	746	92.9 (90.9-94.5)
Unmarried	20	2.5 (1.6-3.8)
Widowed/separated/destitute	37	4.6 (3.4-6.3)
**Education**		
No institutional education	444	55.3 (51.8-58.7)
Primary level (years 1–5)	197	24.5 (21.7-27.6)
Secondary level and above (6 years and above)	162	20.2 (17.5-23.1)
**Occupation**		
Agricultural/labor	143	17.8 (15.3-20.6)
Housewife	519	64.6 (61.3-67.9)
Small business	51	6.4 (4.9-8.3)
Service worker	38	4.7 (3.5-6.4)
Others (e.g., Blacksmiths, students)	52	6.5 (5.0-3.7)
**Having chronic disease in the past 3 months**		
No	731	91.0 (88.8-92.8)
Yes	72	9.0 (7.2-11.2)
**Number of health problems reported**		
No	523	65.1 (61.8-68.4)
One health problem	263	32.8 (29.6-36.1)
Multimorbidity (two or more health problems)	17	2.1 (1.3-3.4)
**Sought healthcare in the last three months**		
No	583	72.6 (69.4-75.6)
Yes	220	27.4 (24.4-30.6)
**Household size**		
Less than 4 persons	298	37.1 (33.8-40.5)
4–5 persons	426	53.1 (49.6-56.5)
6 persons or more	79	9.8 (8.0-12.1)
**Asset quintile**		
Poorest	161	20.0 (17.4-23.0)
Second	161	20.0 (17.4-23.0)
Third	160	19.9 (17.3-22.8)
Fourth	161	20.0 (17.4-23.0)
Richest	160	19.9 (17.3-22.8)

### Distribution of EQ-5D dimensions, EQ-5D score, and VAS score

Table 2 reports the percentage of respondents experiencing problems in each EQ-5D dimension, estimated EQ-5D score, and EQ-VAS score by socioeconomic and demographic characteristics. The BPL population had an average EQ-5D score of 0.738 ± 0.192 and an EQ-VAS score of 0.751 ± 0.149. The results showed that a higher proportion of elderly people (aged 60 years or older) reported problems (some or extreme) in all five dimensions compared to the other age groups (p < 0.001). The elderly also had a lower EQ-5D (0.589 ± 0.225) and VAS score (0.652 ± 0.148) compared to the younger respondents, and there was a statistically significant difference in both scores across the age groups (p < 0.001).

**Table 2:** Percentage of respondents reporting some or extreme problems in each EQ-5D dimension by demographic and socioeconomic characteristics (n = 803).

	Mobility	Self-care	Usual activities	Pain or Discomfort	Anxiety or depression	EQ-5D score	VAS score
	**n (%)**	**n (%)**	**n (%)**	**n (%)**	**n (%)**	**mean (SD)**	**mean (SD)**
**Age group** ^**a), b)**^							
	*p < 0.001*	*p < 0.001*	*p < 0.001*	*p < 0.001*	*p < 0.001*	*p < 0.001*	*p < 0.001*
18-34	20 (7.19)	5 (1.80)	26 (9.35)	107 (38.49)	143 (51.44)	0.811 (0.18)	0.813 (0.14)
35-44	28 (11.52)	2 (0.82)	37 (15.23)	136 (55.97)	167 (68.72)	0.740 (0.19)	0.753 (0.14)
45-60	32 (14.41)	9 (4.05)	62 (27.93)	152 (68.47)	181 (81.53)	0.685 (0.16)	0.700 (0.14)
60+	24 (40.00)	7 (11.67)	27 (45.00)	46 (76.67)	53 (88.33)	0.589 (0.22)	0.652 (0.15)
**Sex** ^**c), d)**^							
	*p = 0.277*	*p = 0.781*	*p = 0.666*	*p = 0.058*	*p = 0.82*	*p = 0.505*	*p = 0.905*
Male	29 (15.26)	6 (3.16)	38 (20.00)	93 (48.95)	130 (68.42)	0.738 (0.20)	0.749 (0.16)
Female	75 (12.23)	17 (2.77)	114 (18.6)	348 (56.77)	414 (67.54)	0.738 (0.19)	0.752 (0.145)
**Marital status** ^**a), b)**^							
	p = 0.102	p = 0.736	p < 0.01	p < 0.001	p < 0.001	p < 0.001	p < 0.001
Married	92 (12.33)	22 (2.95)	136 (18.23)	410 (54.96)	504 (67.56)	0.740 (0.19)	0.754 (0.15)
Unmarried	3 (15.00)	0 (0)	2 (10.00)	3 (15.00)	6 (30.00)	0.884 (0.19)	0.86 (0.18)
Widowed/separated	9 (24.32)	1 (2.70)	14 (37.84)	28 (75.68)	34 (91.89)	0.612 (0.19)	0.649 (0.14)
**Education of household members** ^**a), b)**^							
	*p = 0.066*	*p = 0.066*	*p < 0.001*	*p < 0.001*	*p < 0.001*	*p < 0.001*	*p < 0.001*
No institutional education	68 (15.32)	17 (3.83)	106 (23.87)	297 (66.89)	330 (74.32)	0.701 (0.19)	0.721 (0.14)
Primary level	22 (11.17)	1 (0.51)	27 (13.71)	92 (46.7)	130 (65.99)	0.762 (0.17)	0.775 (0.15)
Secondary level and above	14 (8.64)	5 (3.09)	19 (11.73)	52 (32.1)	84 (51.85)	0.811 (0.20)	0.806 (0.14)
**Occupation** ^**a), b)**^							
	*p = 0.17*	*p < 0.05*	*p = 0.314*	*p < 0.05*	*p = 0.361*	*p = 0.558*	*p = 0.131*
Agricultural/labor	17 (11.89)	2 (1.40)	27 (18.88)	76 (53.15)	100 (69.93)	0.741 (0.167)	0.745 (0.15)
Housewife	63 (12.14)	16 (3.08)	95 (18.3)	303 (58.38)	353 (68.02)	0.735 (0.19)	0.751 (0.14)
Small business	10 (19.61)	0 (0)	12 (23.53)	24 (47.06)	37 (72.55)	0.741 (0.19)	0.775 (0.16)
Service worker	3 (7.89)	0 (0)	4 (10.53)	13 (34.21)	25 (65.79)	0.784 (0.16)	0.795 (0.14)
Others (e.g., Blacksmith)	11 (21.15)	5 (9.62)	14 (26.92)	25 (48.08)	29 (55.77)	0.729 (0.29)	0.725 (0.20)
**Having chronic disease in the past three months** ^**c), d)**^							
	*p < 0.05*	*p < 0.05*	*p < 0.001*	*p < 0.001*	*p < 0.001*	*p < 0.001*	*p < 0.001*
No	89 (12.18)	18 (2.46)	125 (17.1)	386 (52.8)	483 (66.07)	0.748 (0.19)	0.760 (0.15)
Yes	15 (20.83)	5 (6.94)	27 (37.5)	55 (76.39)	61 (84.72)	0.636 (0.21)	0.667 (0.15)
**Number of Health Problem** ^**a), b)**^							
	*p < 0.001*	*p < 0.001*	*p < 0.001*	*p < 0.001*	*p < 0.001*	*p < 0.01*	*p < 0.001*
No	41 (7.84)	8 (1.53)	58 (11.09)	233 (44.55)	316 (60.42)	0.782 (0.17)	0.790 (0.13)
One health problem	54 (20.53)	12 (4.56)	83 (31.56)	192 (73)	213 (80.99)	0.670 (0.19)	0.686 (0.15)
Multimorbidity (two or more health problems)	9 (52.94)	3 (17.65)	11 (64.71)	16 (94.12)	15 (88.24)	0.458 (0.27)	0.588 (0.16)
**Sought healthcare in the last three months** ^**c), d)**^							
	*p < 0.001*	*p < 0.001*	*p < 0.001*	*p < 0.001*	*p < 0.001*	*p < 0.001*	*p < 0.001*
No	51 (8.75)	10 (1.72)	83 (14.24)	280 (48.03)	370 (63.46)	0.770 (0.17)	0.778 (0.14)
Yes	53 (24.09)	13 (5.91)	69 (31.36)	161 (73.18)	174 (79.09)	0.655 (0.21)	0.682 (0.16)
**Household size** ^**a), b)**^							
	*p = 0.994*	*p = 0.97*	*p < 0.05*	*p < 0.001*	*p < 0.01*	*p < 0.001*	*p = 0.19*
Less than 4 persons	39 (13.09)	9 (3.02)	71 (23.83)	184 (61.74)	222 (74.5)	0.715 (0.18)	0.738 (0.16)
4–5 persons	55 (12.91)	12 (2.82)	69 (16.2)	208 (48.83)	270 (63.38)	0.753 (0.20)	0.761 (0.15)
6 persons or more	10 (12.66)	2 (2.53)	12 (15.19)	49 (62.03)	52 (65.82)	0.745 (0.184)	0.753 (0.13)
**Asset quintile** ^**a), b)**^							
	*p < 0.01*	*p < 0.05*	*p < 0.05*	*p = 0.311*	*p = 0.106*	*p < 0.01*	*p = 0.73*
Poorest	31 (19.25)	7 (4.35)	36 (22.36)	96 (59.63)	123 (76.4)	0.701 (0.21)	0.743 (0.17)
Second	26 (16.15)	9 (5.59)	41 (25.47)	95 (59.01)	108 (67.08)	0.710 (0.23)	0.737 (0.16)
Third	11 (6.88)	3 (1.88)	28 (17.5)	87 (54.38)	107 (66.88)	0.743 (0.17)	0.761 (0.13)
Fourth	20 (12.42)	1 (0.62)	21 (13.04)	82 (50.93)	105 (65.22)	0.766 (0.17)	0.755 (0.14)
Richest	16 (10.00)	3 (1.88)	26 (16.25)	81 (50.63)	101 (63.13)	0.77 (0.17)	0.762 (0.15)
**Overall**	**104 (12.95)**	**23 (2.86)**	**152 (18.93)**	**441 (54.92)**	**544 (67.75)**	**0.738 (0.19)**	**0.751 (0.15)**

a) Pearson’s chi-square test and ^b)^ Two sample tests on equality of proportions of columns mobility to anxiety; ^c)^ Rank-sum and Kruskal-Wallis test for equality of means of EQ-5D and VAS scores

Out of all five dimensions of EQ-5D, a significantly higher proportion of female respondents reported problems only in the pain or discomfort (57% vs. 49%) dimension compared to their male counterparts. Statistically significant differences were observed (p < 0.001) in performing usual activities, suffering from pain and discomfort, and having anxiety or depression across marital status and education levels of the respondents. Widowed/separated respondents reported greater difficulties compared to unmarried respondents in performing usual activities (38% vs. 18%), pain or discomfort (75% vs. 55%), and anxiety or depression (92% vs. 68%). Additionally, a significantly higher proportion of respondents with no institutional education reported these issues compared to those with primary or secondary and above levels of education. A significantly (p < 0.05) higher proportion of housewives reported suffering from pain or discomfort compared to respondents from other occupations. The EQ-5D score and VAS score were higher among the service workers than among the respondents from other occupations; however, there was no statistically significant variation in the EQ-5D score and VAS score across the occupations (p > 0.05). A significantly higher proportion of respondents who had chronic disease in the last three months reported experiencing problems in all five dimensions of the EQ-5D. The estimated EQ-5D score and VAS score were also significantly lower among respondents with chronic diseases within the past three months before the survey (p < 0.001). Among the respondents with two or more health problems, a higher proportion reported having problems in all five dimensions of the EQ-5D, and the difference was statistically significant (p < 0.001) across the groups. The EQ-5D score (0.458; p < 0.01) and EQ-VAS score (0.588; p < 0.01) were significantly lower among the respondents with two or more health problems than among those who reported no health problems. A significant variation was observed in pain or discomfort and anxiety or depression by the household size of the respondents. There was a significant variation across income categories in the proportion of respondents who reported experiencing problems in performing usual activities. The proportion of respondents who reported problems across the wealth quintiles also significantly varied in the dimensions of mobility, self-care, and usual activities; however, the direction of proportion remained consistent across the dimensions. EQ-5D scores also varied significantly (p < 0.001) across the wealth quintiles with the EQ-5D score being higher among respondents belonging to the richest quintile. The EQ-5D score also significantly varied across the wealth quintile (p < 0.01), where the first two quintiles had the lowest score compared to the other group.

**Fig 1:**
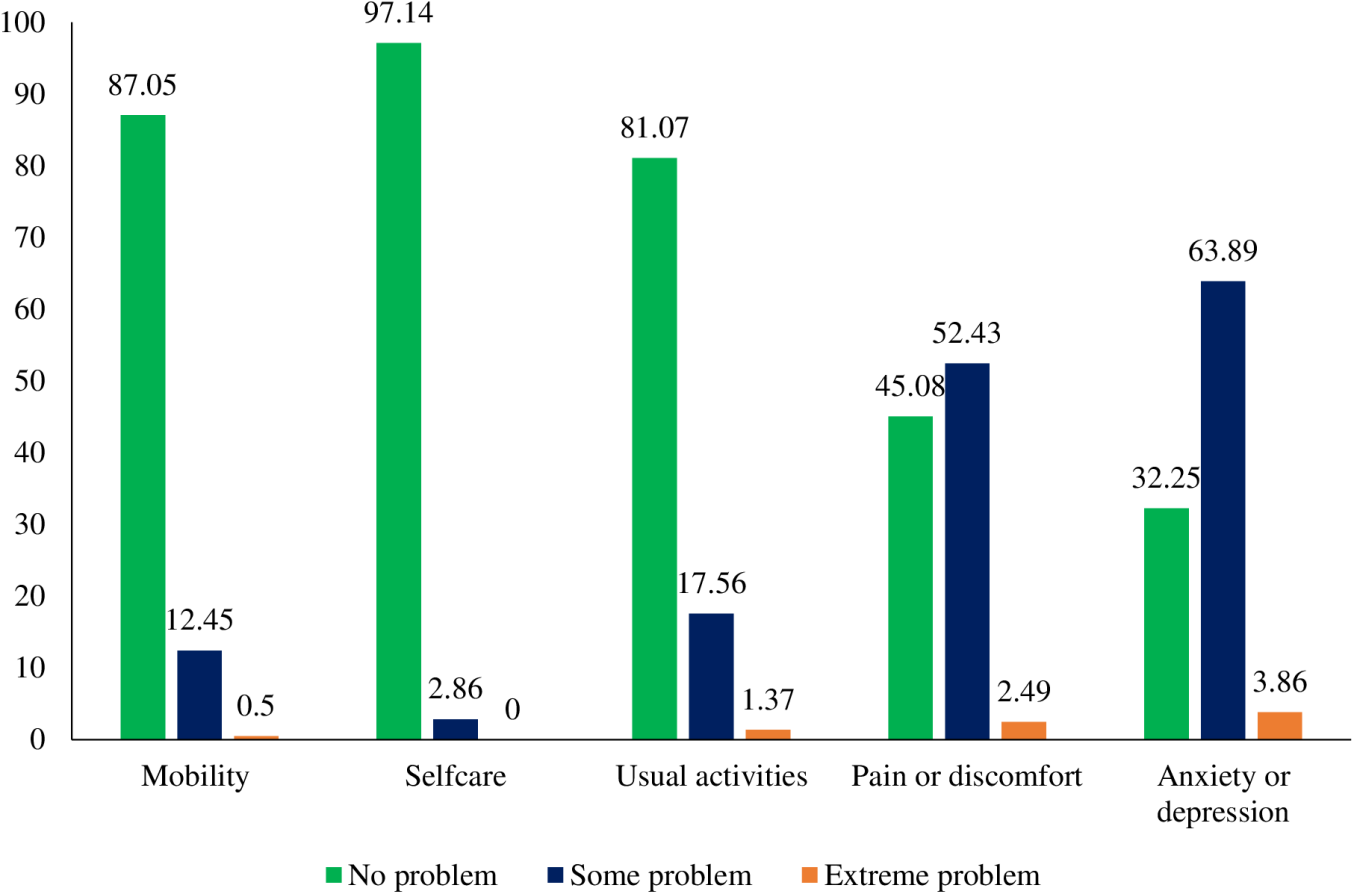
Distribution of EQ-5D responses.

Fig 1 shows the distribution of EQ-5D responses across the five dimensions of the EQ-5D at three levels. Most of the respondents reported having no problems with mobility (87%), self-care (97%), and usual activities (81%). Approximately 55% and 68% of the respondents reported having problems of pain or discomfort and suffering from anxiety or depression, respectively. Of the five dimensions, the highest reported problem was anxiety or depression (3.9%), followed by pain or discomfort (2.5%), usual activities (1.4%), and mobility (0.5%).

### Association of problems in EQ-5D dimensions with background characteristics

Table 3 presents the adjusted odds ratios for reporting problems in the EQ-5D dimensions by respondents’ background characteristics. We found that respondents aged 45 years and above were significantly more likely to report problems in mobility, usual activities, and pain or discomfort dimensions compared to respondents aged between 18–34 years. However, only respondents aged 60 or older were significantly more likely to report problems in self-care (OR=11.07; p < 0.001) compared to respondents aged between 18–34 years. Similarly, respondents from all other age groups were significantly more likely to report problems in the anxiety or depression dimension. Unmarried respondents were significantly more likely to report mobility problems than married respondents (OR=4.54; p < 0.05). No statistically significant association was observed between the occupation of respondents and EQ-5D dimensions, except for anxiety or depression. Respondents with two or more health problems were significantly more likely to report problems in mobility (OR=5.44; p < 0.05), usual activities (OR=12.98; p < 0.001), and pain or discomfort (OR=14.19; p < 0.05) dimensions than respondents with no reported health problems. Respondents with one health problem were significantly more likely to report problems in performing usual activities (OR=4.50; p < 0.001) and experiencing pain or discomfort (OR=3.50; p < 0.01) and anxiety or depression (OR=4.53; p < 0.01) compared to respondents with no problems. The likelihood of reporting pain or discomfort was significantly lower among respondents from 4-5-member households (OR=0.70: p < 0.05) than among respondents from households with fewer than 4 members. Although the respondents belonging to the richest wealth quintile had the lowest odds of reporting problems in any dimension, these were only statistically significant for the anxiety or depression dimension (OR=0.57; p < 0.05).

**Table 3:** Association of health problems reported in different dimensions of EQ-5D with the background characteristics of the respondents (n = 803).

Characteristics	Mobility	Self-care	Usual activities	Pain or Discomfort	Anxiety or depression
Adjusted OR (95% CI)	Adjusted OR (95% CI)	Adjusted OR (95% CI)	Adjusted OR (95% CI)	Adjusted OR (95% CI)
**Age group**					
18-34	**Ref.**	**Ref.**	**Ref.**	**Ref.**	**Ref.**
35-44	1.819 (0.93,3.56)	0.398 (0.07,2.37)	1.570 (0.87,2.84)	1.360 (0.91,2.04)	1.736** (1.16,2.61)
45-60	2.676* (1.27,5.66)	4.445 (0.98,20.26)	3.654*** (1.93,6.92)	2.056** (1.27,3.33)	3.427*** (2.05,5.74)
60+	11.070*** (4.13,29.69)	12.620* (1.65,96.31)	8.008*** (3.28,19.54)	3.247** (1.43,7.39)	6.995*** (2.56,19.10)
**Sex**					
Male	**Ref.**	**Ref.**	**Ref.**	**Ref.**	**Ref.**
Female	1.536 (0.63,3.76)	3.735 (0.23,59.45)	1.595 (0.73,3.49)	1.065 (0.57,1.98)	0.927 (0.49,1.76)
**Marital status**					
Married	**Ref.**	**Ref.**	**Ref.**	**Ref.**	**Ref.**
Unmarried	4.544* (1.01,20.42)	1 (1.00, 1.00)	1.503 (0.28,7.93)	0.342 (0.08,1.40)	0.645 (0.20,2.10)
Widowed/separated/destitute	1.191 (0.45,3.15)	0.245 (0.02,3.70)	1.636 (0.70,3.81)	1.683 (0.72,3.95)	3.686* (1.03,13.21)
**Education**					
No institutional education	**Ref.**	**Ref.**	**Ref.**	**Ref.**	**Ref.**
Primary level (years 1–5)	1.147 (0.63,2.09)	0.229 (0.03,2.01)	0.838 (0.49,1.44)	0.555** (0.37,0.83)	1.071 (0.70,1.63)
Secondary level and above	1.051 (0.49,2.23)	3.483 (0.82,14.78)	1.054 (0.55,2.02)	0.374*** (0.23,0.60)	0.802 (0.50,1.28)
**Occupation**					
Agricultural/labor	**Ref.**	**Ref.**	**Ref.**	**Ref.**	**Ref.**
Housewife	1.360 (0.59,3.11)	1.989 (0.20,20.20)	1.289 (0.65,2.57)	1.975* (1.14,3.42)	1.587 (0.90,2.80)
Small business	2.841* (1.05,7.66)	**–**	1.719 (0.70,4.24)	1.339 (0.64,2.82)	1.978 (0.90,4.37)
Service worker	0.881 (0.21,3.66)	**–**	0.934 (0.27,3.29)	0.806 (0.34,1.93)	1.446 (0.61,3.43)
Others (e.g., Blacksmith, students)	1.312 (0.47,3.70)	4.990 (0.70,35.43)	1.535 (0.59,3.97)	1.416 (0.60,3.34)	0.696 (0.29,1.67)
**Having chronic disease in the past three months**					
No	**Ref.**	**Ref.**	**Ref.**	**Ref.**	**Ref.**
Yes	0.970 (0.47,2.01)	2.042 (0.57,7.34)	1.079 (0.57,2.04)	1.070 (0.54,2.14)	1.206 (0.55,2.64)
**Number of Health Problem**					
No	**Ref.**	**Ref.**	**Ref.**	**Ref.**	**Ref.**
One health problem	1.985 (0.83,4.78)	0.908 (0.13,6.58)	4.496*** (2.23,9.07)	3.496** (1.65,7.42)	4.529** (1.75,11.71)
Multimorbidity (two or more health problems)	5.438* (1.35,21.96)	4.517 (0.37,54.75)	12.98*** (3.59,46.95)	14.19* (1.62,124.00)	4.017 (0.70,23.12)
**Sought healthcare in the last three months**					
No	**Ref.**	**Ref.**	**Ref.**	**Ref.**	**Ref.**
Yes	1.674 (0.73,3.84)	2.872 (0.47,17.56)	0.644 (0.33,1.26)	0.827 (0.39,1.75)	0.452 (0.18,1.17)
**Household size**					
Less than 4 persons	**Ref.**	**Ref.**	**Ref.**	**Ref.**	**Ref.**
4–5 persons	1.599 (0.96,2.65)	1.847 (0.63,5.41)	0.866 (0.57,1.32)	0.695* (0.49,0.98)	0.740 (0.52,1.06)
6 persons or more	1.428 (0.64,3.21)	1.551 (0.27,8.88)	0.648 (0.31,1.35)	1.218 (0.68,2.17)	0.676 (0.37,1.22)
**Asset quintile**					
Poorest	**Ref.**	**Ref.**	**Ref.**	**Ref.**	**Ref.**
Second	0.995 (0.52,1.91)	2.227 (0.64,7.79)	1.679 (0.92,3.06)	1.119 (0.67,1.86)	0.699 (0.41,1.19)
Third	0.334** (0.15,0.74)	0.517 (0.11,2.46)	1.034 (0.56,1.92)	1.003 (0.61,1.66)	0.726 (0.43,1.24)
Fourth	0.733 (0.37,1.45)	0.146 (0.02,1.36)	0.656 (0.34,1.27)	0.791 (0.48,1.31)	0.567* (0.33,0.97)
Richest	0.500 (0.24,1.04)	0.457 (0.10,2.18)	0.861 (0.45,1.65)	0.901 (0.53,1.52)	0.572* (0.33,0.99)
Constant	0.022*** (0.01,0.07)	0.001*** (0.00,0.02)	0.037*** (0.01,0.10)	0.607 (0.29,1.26)	1.218 (0.56,2.63)
n	803.00	696.00	803.00	803.00	803.00
Log likelihood	-264.20	-75.84	-326.90	-466.90	-442.20
Chi-square	90.73	50.40	125.40	171.50	125.50
p value	p < 0.001	p < 0.001	p < 0.001	p < 0.001	p < 0.001
Pseudo-R-square	0.15	0.25	0.16	0.16	0.12

* p < 0.05, **p < 0.01, *** p < 0.001.

### Determinants of EQ-5D and VAS scores

Table 4 presents the adjusted coefficients from two right-censored Tobit regression models where the dependent variables were (1) estimated EQ-5D scores and (2) EQ-VAS scores. We found that compared to younger adults aged 18–34 years, EQ-5D scores were significantly (p < 0.001) lower among the higher age groups as evidenced by negative coefficients. EQ-5D scores were lower by 0.069 points for individuals aged 35–44 years, 0.137 points for 45–60 years, and 0.218 points for 60 years and above. A similar association (p < 0.001) with the age of the respondents was observed in the EQ-VAS scores. The predicted value of the EQ-VAS score was 0.079 (p < 0.05) points higher for unmarried respondents and 0.069 (p < 0.01) points lower for widowed/separated respondents compared to married respondents. Similar relationships were predicted for the EQ-5D score, although were not statistically significant (p > 0.05). Both the EQ-5D scores and EQ-VAS scores were statistically significantly higher for respondents with secondary and above levels of education by 0.061 (p < 0.01) points and 0.032 (p < 0.05) points, respectively. EQ-5D and VAS scores were predicted to be lower among the respondents with chronic disease, however, this difference was not statistically significant. Compared to no health problems, both EQ-5D and EQ-VAS scores were predicted to be statistically significantly lower for respondents having one health problem and respondents having multiple health problems; 0.087 (p < 0.01) and 0.241 (p < 0.001) points lower in EQ-5D scores; and 0.090 (p < 0.001) and 0.150 (p < 0.001) points lower in EQ-VAS scores. The respondents belonging to the richest wealth quintile had higher EQ-5D scores and EQ-VAS scores than the respondents belonging to the poorest wealth quintile; however, the difference was only statistically significant for the EQ-5D score. Compared to agriculture workers, housewives had lower EQ-5D and EQ-VAS scores, but the relation was statistically significant for the EQ-5D score only. Sex, household size, and healthcare-seeking behaviour in the last three months were not significantly associated with the EQ-5D and EQ-VAS scores.

**Table 4:** Factors associated with EQ-5D index and VAS score.

	Dependent = EQ-5D score Coefficient (95% CI)	Dependent = VAS score Coefficient (95% CI)
**Age group**		
18-34	**Ref.**	**Ref.**
35-44	-0.069** (-0.11, -0.03)	-0.046*** (-0.07, -0.02)
45-60	-0.137*** (-0.18, -0.09)	-0.093*** (-0.12, -0.06)
60+	-0.218*** (-0.29, -0.14)	-0.124*** (-0.17, -0.08)
**Sex**		
Male	**Ref.**	**Ref.**
Female	-0.002 (-0.06,0.06)	-0.006 (-0.04,0.03)
**Marital status**		
Married	**Ref.**	**Ref.**
Unmarried	0.113 (-0.01,0.24)	0.079* (0.00,0.16)
Widowed/separated	-0.079* (-0.15, -0.00)	-0.069** (-0.12, -0.02)
**Education**		
No institutional education	**Ref.**	**Ref.**
Primary level (years 1–5)	0.012 (-0.03,0.05)	0.023 (-0.00,0.05)
Secondary level and above	0.042 (-0.01,0.09)	0.027 (-0.00,0.06)
**Occupation**		
Agricultural/labor	**Ref.**	**Ref.**
Housewife	-0.059* (-0.11, -0.01)	-0.0245 (-0.06,0.01)
Small business	-0.037 (-0.11,0.04)	0.006 (-0.04,0.05)
Service worker	-0.017 (-0.10,0.07)	0.009 (-0.04,0.06)
Others (e.g., Blacksmiths, students)	-0.015 (-0.09,0.06)	-0.045 (-0.10,0.01)
**Having chronic disease in the past three months**		
No	**Ref.**	**Ref.**
Yes	-0.032 (-0.09,0.03)	-0.00832 (-0.05,0.03)
**Number of health problems**		
No	**Ref.**	**Ref.**
One health problem	-0.088** (-0.15, -0.02)	-0.090*** (-0.13, -0.05)
Multimorbidity (two or more health problems)	-0.241*** (-0.36, -0.12)	-0.150*** (-0.23, -0.07)
**Sought healthcare in the last three months**		
No	**Ref.**	**Ref.**
Yes	-0.022 (-0.09,0.04)	0.004 (-0.04,0.04)
**Household size**		
Less than 4 persons	**Ref.**	**Ref.**
4–5 persons	0.013 (-0.02,0.05)	-0.004 (-0.03,0.02)
6 persons or more	0.019 (-0.04,0.07)	0.005 (-0.03,0.04)
**Asset quintile**		
Poorest	**Ref.**	**Ref.**
Second	-0.014 (-0.06,0.03)	-0.027 (-0.06,0.00)
Third	0.018 (-0.03,0.07)	-0.006 (-0.04,0.03)
Fourth	0.059* (0.01,0.11)	-0.007 (-0.04,0.02)
Richest	0.057* (0.01,0.11)	0.002 (-0.03,0.03)
Constant	0.888*** (0.82,0.96)	0.862*** (0.82,0.91)
Sigma	0.046*** (0.04,0.05)	0.019*** (0.02,0.02)
n	803.00	803.00
Log likelihood	-109.70	335.80
Chi-square	211.50	225.20
p value	p < 0.001	p < 0.001
Pseudo-R-square	0.49	-0.50

* p < 0.05, **p < 0.01, *** p < 0.001.

## Discussion

This study measured HRQoL among BPL individuals and identified associated factors. We found that elderly individuals compared to younger individuals, and widowed/separated individuals compared to married individuals, had statistically significantly lower HRQoL. Having one or multiple morbidities significantly reduced the HRQoL of the respondents in comparison to those without any morbidities. The respondents in the richest wealth quintile had higher EQ-5D scores compared to those who belonged to the poorest quintile.

Across the dimensions of EQ-5D, compared to younger respondents, those aged 60 and older were statistically significantly more likely to experience difficulties in the mobility, self-care, usual activities, and pain or discomfort dimensions. The overall EQ-5D score also statistically significantly decreased with the increase in age of the respondents from 35 years to 60 years or older, compared to the respondents aged between 18–34 years. A similar association with the age of the respondents was observed in the EQ-VAS scores. Higher odds of poor HRQoL among the elderly population have been observed in previous research [14]. Many factors have been identified to contribute to this linkage between age and reduced HRQoL in empirical research, such as the risk of chronic illness, geriatric diseases, and lack of financial support [32]. In particular, the significant difficulties reported in the self-care dimension highlighted the increasing dependency of older adults on caregivers for basic daily activities, which might be influenced by limited access to elderly-specific healthcare services and social support systems in Bangladesh. Moreover, as people grow older, they generally experience reduced mobility, increased difficulty in self-care, and a higher likelihood of developing age-related conditions such as cognitive or functional impairment [33].

We also found that the EQ-5D and EQ-VAS scores were statistically significantly lower among the widowed/separated respondents and were higher among the unmarried respondents compared to the married respondents. A study in Korea showed a significant relationship between marital status and HRQoL and that relationship appeared to be similar to our study [34]. Another study in China reported that widowed individuals had poorer physical and mental quality of life than their married counterparts [35]. Such disparities may be partially explained by cultural and social factors, as reported in a previous literature [36]. In the context of Bangladesh, social norms and expectations surrounding marital status can influence both the actual health status and the way individuals report their health. Widowed or separated women, in particular, may face economic insecurity, social isolation, and reduced access to healthcare, all of which can contribute to poorer HRQoL. Additionally, cultural stigma associated with mental health issues or self-care difficulties could lead to underreporting, shaping the observed patterns in HRQoL. We also found that unmarried respondents were significantly more likely to report mobility problems than married respondents. In addition to cultural factors, existing illness could be another possible explanation. Among the twenty unmarried respondents, only three reported mobility problems, and two of them had experienced an illness, suggesting that existing morbidity may be a key driver of this finding.

Our findings reported that people with a higher level of education had a higher HRQoL than those with no institutional education. However, the findings were not statistically significant. A study conducted in Malaysia reported that individuals with a lower level of education had a lower HRQoL than their peers with a higher level of education [37]. Better education leads to a greater understanding of health issues and better health choices, which consequently results in a better quality of life [38]. One possible reason for the non-significant association in our study could be the relatively homogenous sociodemographic characteristics of the BPL population, including education levels, which may have limited variability in the data. Our findings also showed that a higher proportion of respondents with chronic illness reported problems with all dimensions of the EQ-5D and had lower HRQoL. However, the association was not statistically significant in the adjusted regression model. Previous studies demonstrated that the presence of a chronic medical condition had a substantial impact on HRQoL owing to the constraints imposed by the disease or emergency issues [39,40].

We found that respondents with one or more health problems were more likely to report problems in different dimensions of the EQ-5D. The predicted EQ-5D and EQ-VAS scores for these respondents were also significantly lower compared to the scores of respondents with no health problems. A study in Bangladesh reported that the presence of single or multiple morbidities was likely to reduce HRQoL and was inversely proportional to the number of comorbidities [16]. We found that respondents from the richest wealth quintile were less likely to report any health problems than respondents from the poorest quintile. Similar findings were reported in the literature indicating that people with lower socioeconomic status have increased levels of health impairments and lower HRQoL [39–41].

BPL individuals are at a higher risk of experiencing poor HRQoL, especially when they are older and have one or multiple health problems. These individuals often have limited access to healthcare, nutritious food, and safe living conditions, all of which can contribute to poor health [42,43]. Access to healthcare is also limited for individuals living below the poverty line, as they may not have the financial capacity to visit a doctor or purchase medication in time, which can lead to poor health conditions. This can also lead to a cycle of poverty, as individuals are unable to work due to their poor health, which in turn makes it harder for them to escape poverty [44]. To address such a situation, the BPL population may require multidimensional interventions focusing on healthcare, as reflected in the 2011 National Health Policy [45]. The 2012 healthcare financing strategy of the government of Bangladesh also proposed several approaches for different groups of populations to improve their access to healthcare services, aiming to achieve universal health coverage by 2032 [46]. Based on this, a health protection scheme is being implemented to improve access of the BPL population to inpatient healthcare services [23,47]. The successful implementation of such initiatives across the country may increase access to healthcare services that the BPL population needs and help improve their HRQoL. Additionally, our findings highlight the need to focus on elderly care, as older people, especially those with multimorbidity, exhibited significantly lower HRQoL. Despite policy efforts to expand service access for BPL populations, limited attention has been given to elderly care in Bangladesh. Introducing community-based elder care programs and chronic disease management services could improve the quality of life among older adults. Additionally, the identification and enrolment of BPL households should be refined to ensure inclusion of socioeconomically and socially vulnerable subgroups, such as widowed or separated individuals, who may experience both economic hardship and social isolation, factors likely contributing to their poorer health outcomes.

### Strengths and limitations

This study contributed to the existing literature by generating evidence on the HRQoL of the BPL population, which is the first of its kind in Bangladesh. Findings can be considered as a baseline for evaluating interventions aimed at improving the HRQoL of the BPL population in Bangladesh, particularly the effect of the health protection scheme.

This study is subject to certain limitations. The cross-sectional design of this study limits us from establishing a causal association between identified factors and HRQoL. The study was conducted in a particular rural area and involved a random sample of BPL households from multiple communities; hence, its findings cannot be generalized to the entire population of Bangladesh. The sample was predominantly female, with a large proportion reporting no chronic disease, no recent healthcare-seeking in the past three months, and no current health problems. These characteristics may reflect the demographic and health profile of the surveyed BPL group, but they could also introduce bias in estimating overall HRQoL. The survey was conducted in 2018, and since then, policy-driven changes and socioeconomic developments may have taken place, particularly affecting the BPL population. Thus, the findings should be interpreted cautiously as these changes could influence income levels, healthcare access, and living conditions, thereby affecting HRQoL. Another limitation of our study is that we used a Bengali-translated version of the EQ-5D questionnaire, which is not validated. While we have checked the internal consistency using Cronbach’s alpha, this metric may not be optimal for evaluating EQ-5D, which captures distinct health domains. Future studies can conduct psychometric testing, such as assessing construct validity, responsiveness, or test-retest reliability, to strengthen confidence in the instrument’s appropriateness for this context. Additionally, due to the absence of country-specific value sets for Bangladesh, we used the Thailand-based tariff to estimate the EQ-5D scores. While this approach has been used in several other studies [48,49], it may not fully capture local population norms. Although the study population was enrolled in a pilot health protection scheme, the survey was conducted during its initial phase. Therefore, the influence of the scheme on HRQoL or health-seeking behaviour is likely minimal.

## Conclusions

The findings of this study highlighted that several factors influenced the HRQoL of the BPL population, including age, multimorbidity, marital status, and wealth status. The BPL population experiences complex socioeconomic challenges, including access to quality healthcare services. Tailored interventions are needed to address the specific needs for improving their HRQoL such as community-based programs for the elderly, provision for integrated healthcare to address multimorbidity, and the effective implementation of the current health protection scheme for the poor and other vulnerable groups, e.g., widowed/separated individuals. Future studies may compare the determinants of HRQoL between the general population and the BPL population to inform more equitable and inclusive policy development.

## Supporting information

S1 Data Dataset used in this mansucript. (XLS)

S1 Checklist Inclusivity in global research. (DOCX)
